# Efficacy of PRP versus hyaluronic acid in obese individuals with knee osteoarthritis: a retrospective study

**DOI:** 10.1007/s00590-026-04922-8

**Published:** 2026-08-12

**Authors:** Aleksi Annaniemi, Jüri Pere, Salvatore Giordano

**Affiliations:** 1 Department of Surgery, Satasairaala Hospital, Pori, Finland; 2 Department of Clinical Medicine, University of Turku, Turku, Finland; 3 Department of Surgery, Satasairaala Hospital, Pori, Finland

**Keywords:** Obesity, Platelet-rich plasma, Hyaluronic acid, Knee osteoarthritis, Arthroplasty

## Abstract

**Introduction:**

Obesity is a common comorbidity in patients with knee osteoarthritis (KOA). Both viscosupplementation and platelet-rich plasma (PRP) intra-articular injections may alleviate KOA symptoms. To better guide patient selection for PRP therapy, we compared the outcomes of these treatments in obese individuals.

**Materials and methods:**

This retrospective study included 99 obese patients who received intra-articular injections of either PRP (*n* = 43) or hyaluronic acid (HA, *n* = 56) between January 2014 and October 2017. The Western Ontario and McMaster Universities Osteoarthritis Index (WOMAC), visual analogue scale (VAS), and range of motion (ROM) were assessed before the injection and at 15 days, 6 months, 12 months, and the final follow-up. Outcomes were then compared between the two treatment groups.

**Results:**

Obese patients treated with HA had a higher rate of arthroplasty (35.7% vs. 9.3%, *p* = 0.002), reduced ROM, poorer VAS and WOMAC scores, and a higher overall risk of arthroplasty (log-rank *p* = 0.177) compared with those treated with PRP. Cox proportional hazards analysis indicated a non-significant trend toward a reduced risk of knee arthroplasty in the PRP group (HR = 0.46, 95% CI 0.15–1.42, *p* = 0.176) over average follow-up of 16.5 month for all patients.

**Conclusions:**

Intra-articular PRP injections were associated with better outcomes than HA in obese patients with KOA. PRP may delay the need for arthroplasty and could serve as a valuable therapeutic option for selected patients who do not respond to conventional treatments. Due to several limitations, further large-scale studies are warranted to confirm the effectiveness of this promising approach in this high-risk population.

## Introduction

The global burden of knee osteoarthritis (KOA) is rising steadily, while suitable conservative treatment modalities remain largely unchanged and include physical therapy, weight loss, non-steroidal anti-inflammatory drugs, and intra-articular injections of corticosteroids, hyaluronic acid, platelet-rich plasma, and similar biological therapies [1]. Obesity is a major risk factor for KOA, with body mass index (BMI) correlating strongly with decreases in patient-reported outcome measures (PROMs), especially pain and function scores [2]. Weight loss is an effective strategy for treating KOA symptoms and slowing disease progression [2–4]. Despite increasing research focus on targeting obesity as a cornerstone of KOA treatment, not all patients achieve sufficient weight loss to experience clinical benefits. Therefore, studies identifying optimal treatments for obese KOA patients are warranted [2, 4].

Intra-articular platelet-rich plasma (PRP) injections have emerged as a promising treatment for osteoarthritis and are often compared to hyaluronic acid (HA), with studies demonstrating superior results in various settings [5, 6]. A meta-analysis suggests that PRP may be superior to HA at 6- and 12-month time points in relieving pain and improving joint function [7]. Current literature lacks adequate studies on time to arthroplasty, with only a few reports available, albeit with promising results [8, 9]. A clinically important aspect of conservative treatment is not only delaying arthroplasty as long as possible to avoid complications and patient dissatisfaction but also providing adequate symptom relief [10]. The most recent meta-analysis of randomized controlled trials found that PRP demonstrated superior functional improvement compared to HA, physical therapy (PT), and exercise therapy (ET), as well as superior pain management compared to PT and ET. However, no significant difference was observed in monotherapy when compared to corticosteroids [10]. Notably, the best effectiveness may be achieved in patients with mild to moderate KOA and when PRP is combined with HA [10].

Obese KOA patients may benefit more from PRP injection therapy than from HA injections [7]. Although debatable, obesity increases the risks associated with knee arthroplasty, and patients with BMI ≥ 35 experience worse functional and biomechanical outcomes than those with BMI < 35 [11]. Even studies of robotic-assisted total knee arthroplasty have found that patients with BMI ≥ 35 have higher complication rates than those with BMI < 35 [12]. Therefore, it is beneficial to offer optimal treatments to obese KOA patients during the early stages of disease and attempt to postpone arthroplasty until appropriate lifestyle modifications can be made to achieve weight loss.

This study focuses on guiding patient selection for injection therapy. The aim of this study is to compare the outcomes of PRP and HA treatments, specifically in obese individuals to determine which option may be more optimal for this patient population.

## Materials and methods

This retrospective study included 99 obese patients who received intra-articular injections of either PRP (*n* = 43) or hyaluronic acid (HA, *n* = 56) between January 2014 and October 2017 at a single institution, performed by a single senior author. The study adhered to the ethical principles of the World Medical Association Declaration of Helsinki and was approved by the Institutional Review Board. Source data were collected retrospectively and de-identified; therefore, individual informed consent was waived.

Patients were divided into two groups (A and B). Group A received intra-articular PRP injection therapy (*n* = 43), and Group B received intra-articular HA (*n* = 56). Group A received a total of three injections of autologous PRP manufactured with a commercial Glo PRP kit (GloFinn Corporation, Salo, Finland), while Group B received a single 6-mL injection of high molecular weight HA (Synvisc-One, hylan G-F 20, Sanofi-Aventis, Paris, France). PRP preparation began with an experienced nurse drawing up to 30 mL of venous blood, which was then centrifuged twice. Sodium citrate was used as an anticoagulant to prevent platelet aggregation and clotting. The first centrifugation lasted 5 min at 1200 rpm; excess red blood cells were then removed, and the product was centrifuged a second time for 10 min at 1200 rpm. The final platelet concentrate consisted of 4 to 8 times the physiological normal values, and the white blood cell concentration was doubled according to the manufacturer. The white blood cell levels surpassed baseline levels, meaning that the final product is categorized as leukocyte-rich PRP. The volume of the final product administered into the joint space ranged from 4 to 5 mL. Both PRP and HA injections were performed by an experienced orthopedic surgeon using aspiration and anatomical landmarks to access joint space. Due to the retrospective nature of the study, comprehensive data or analyses of precise platelet-to-white blood cell ratios in the final PRP product were unattainable. Regarding patient and treatment selection, this retrospective study included patients who had previously received injection treatment. Treatment allocation was not randomized; patients were presented with two treatment options, platelet-rich plasma (PRP) or hyaluronic acid (HA), and selected their preferred treatment.

Demographic data was collected from patients’ electronic medical records. Inclusion criteria were as follows: Visual Analogue Scale (VAS) for pain ranging from 30 to 100 before treatment, mild to moderate KOA on radiographs (Kellgren-Lawrence grade 1 to 3), BMI ≥ 30 and age between 18 and 90 years. Exclusion criteria included major systemic diseases or infections (such as active fulminant rheumatoid disease, immunodeficiency, or hematological disease), clinically relevant hip osteoarthritis on the ipsilateral side, pregnancy or possibility of pregnancy, and age below 18 or above 90 years. The study population represents a typical KOA population with symptomatic mild to moderate KOA, with obesity as the distinguishing characteristic. This may enhance the generalizability of the study results to clinical practice.

The primary outcome measure was the Western Ontario and McMaster Universities Osteoarthritis Index (WOMAC), and secondary outcome measures were Visual Analogue Scale (VAS), range of motion (ROM), and risk of arthroplasty. ROM was recorded using the institutional goniometric system, in which values approaching 90° represent full extension. Patient-reported outcome measures (PROMs) were documented during routine clinical follow-up visits according to institutional practice and were collected retrospectively from electronic medical records. Only patients with sufficient follow-up data available in the medical records were included in the analysis. The number of adverse events in both groups was also documented. WOMAC, VAS, and ROM were assessed before the injection and subsequently at 15 days, 6 months, 12 months, and at the final follow-up. Outcomes were then compared between the two treatment groups. The indication for arthroplasty was determined at the surgeon’s discretion if any patient failed prolonged conservative treatment (typically 6 months) and demonstrated progression of KOA to a more severe Kellgren-Lawrence (KL) grade.

### Statistical analysis

Statistical analyses were performed using SPSS statistical software (IBM SPSS Statistics, version 31, Armonk, NY, USA). Continuous variables are presented as mean ± standard deviation. Normality of data distribution was assessed using histograms, kurtosis, skewness, and Kolmogorov–Smirnov tests when appropriate. Univariate comparisons between the two treatment groups were conducted using Pearson’s chi-square test, Fisher’s exact test, and independent samples t-test, as appropriate. A two-sided p-value of less than 0.05 was considered statistically significant.

## Results

The baseline characteristics and demographics were similar between both groups, with the only differences being mean age (Group A: 56.6 ± 9.9 years vs. Group B: 63.8 ± 9.4 years, *p* < 0.001) and follow-up time in months (Group A: 13.8 ± 4.4 vs. Group B: 18.6 ± 7.3, *p* < 0.001). Comprehensive demographic and baseline data for PROMs are listed in Table 1. The severity of KOA, BMI, and PROM scores were similar in both groups at baseline. Group A (treated with PRP) showed statistically significant improvement in PROMs compared to Group B (treated with HA). VAS for pain was lower in Group A at 6 months, 12 months, and last follow-up. Similarly, overall WOMAC scores were lower in Group A compared to Group B at 6 months, 12 months, and last follow-up. The outcomes during the follow-up period are listed in Table 2. Group A demonstrated better knee extension ROM at 6 months, 12 months, and last follow-up compared to Group B (Table 2).

**Table 1:** Baseline characteristics of obese patients who underwent intra-articular injections of PRP or HA for knee OA

	PRP group *n* = 43	HA group *n* = 56	*p-*value
Mean age (years)	56.6 ± 9.9	63.8 ± 9.4	< 0.001
Females	30 (69.7%)	36 (64.3%)	0.566
Mean BMI^1^ (kg/m^2^)	32.9 ± 3.7	33.2 ± 4.2	0.687
Comorbidity	18 (41.18%)	27 (48.2%)	0.529
Diabetes	6 (13.9%)	11 (19.6%)	0.457
Hypertension	19 (44.2%)	35 (62.5%)	0.070
Cardiac disease	5 (11.6%)	8 (14.3%)	0.698
Lung disease	6 (13.9%)	8 (14.3%)	0.962
Rheumatic disease	6 (13.9%)	4 (7.1%)	0.256
Smokers	10 (23.2%)	4 (7.1%)	0.039
Osteoarthritis Grade (Kellgren-Lawrence, mean ± SD†	2.35 ± 0.6	2.21 ± 0.6	0.299
Osteoarthritis Grade (Kellgren-Lawrence) I	3 (7.0%)	3 (5.3%)	1.000
Osteoarthritis Grade (Kellgren-Lawrence) II	25 (58.1%)	28 (50.0%)	0.849
Osteoarthritis Grade (Kellgren-Lawrence) III	15 (34.9%)	20 (35.7%)	1.000
Osteoarthritis Grade (Kellgren-Lawrence) IV	0 (0.0%)	5 (8.9%)	0.067
Bilateral	5 (11.6%)	7 (12.3%)	0.895
Flexion degree	128.5 ± 21.3	125.8 ± 20.9	0.535
Extension degree	88.8 ± 3.1	88.8 ± 7.9	0.974
VAS^2^ pain score (0–100)	64.5 ± 15.2	66.7 ± 19.9	0.560
WOMAC^3^ overall	32.8 ± 13.8	37.9 ± 15.2	0.105
Follow-up (months)	13.8 ± 4.4	18.6 ± 7.3	< 0.001

^1^BMI; body mass index

^2^VAS; Visual analogue scale

^3^WOMAC; Western Ontario and McMaster Universities Osteoarthritis Index

**Table 2:** Outcomes of obese patients who underwent intra-articular injections of PRP or HA for knee OA

	PRP group *n* = 43	HA group *n* = 56	*p-*value
Adverse events	2 (4.6%)	3 (5.3%)	1.000
Number of injections	3.1 ± 0.7	1.7 ± 1.0	< 0.001
Flexion degree (15 days)	132.6 ± 17.7	129.3 ± 19.0	0.398
Extension degree (15 days)	89.1 ± 2.5	87.9 ± 3.0	0.044
VAS^1^ pain score (0–100)—(15 days)	37.5 ± 21.4	36.0 ± 25.0	0.760
WOMAC^2^ overall (15 days)	21.3 ± 13.1	25.4 ± 15.8	0.194
Flexion degree (6 months)	136.7 ± 15.3	130.5 ± 18.0	0.091
Extension degree (6 months)	89.7 ± 1.1	88.2 ± 2.8	0.002
VAS pain score (0–100)—(6 months)	19.0 ± 22.8	43.6 ± 31.0	< 0.001
WOMAC overall (6 months)	12.7 ± 13.5	27.8 ± 18.4	< 0.001
Flexion degree (12 months)	136.2 ± 15.9	130.1 ± 19.6	0.149
Extension degree (12 months)	89.2 ± 2.2	87.7 ± 3.3	0.022
VAS pain score (0–100)—(12 months)	20.9 ± 26.0	43.6 ± 28.2	< 0.001
WOMAC overall (12 months)	13.6 ± 16.0	29.5 ± 19.9	< 0.001
Flexion degree (last follow-up)	136.0 ± 16.2	128.5 ± 20.2	0.060
Extension degree (last follow-up)	89.0 ± 2.6	87.5 ± 3.4	0.027
VAS pain score (0–100)—(last follow-up)	24.2 ± 28.4	55.0 ± 27.7	< 0.001
WOMAC overall last follow-up)	15.6 ± 17.3	35.0 ± 20.7	< 0.001
Any knee arthroplasty	4 (9.3%)	20 (35.7%)	0.002
Unicompartmental knee arthroplasty	2 (4.6%)	10 (17.8%)	0.063
Total knee arthroplasty	2 (4.6%)	10 (17.8%)	0.063

^1^VAS; Visual analogue scale

^2^WOMAC; Western Ontario and McMaster Universities Osteoarthritis Index

**Fig. 1:**
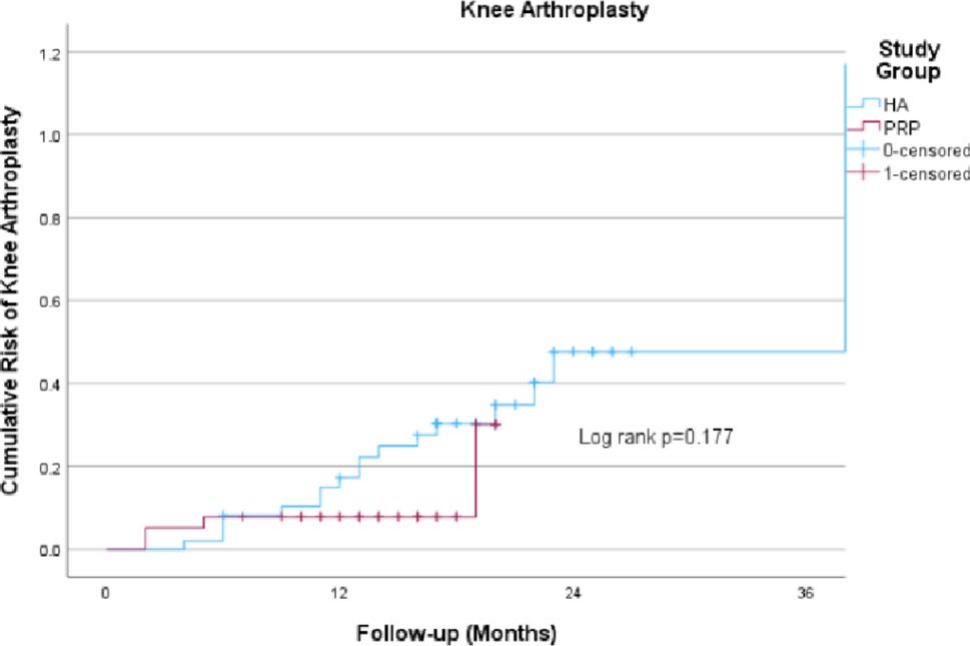
Cumulative risk of any knee arthroplasty during the follow-up between Group A and Group B

A significant difference in the number of arthroplasties was detected between the groups [Group A: 4 (9.3%) vs. Group B: 20 (35.7%), *p* = 0.002] (Table 2). The cumulative risk of knee arthroplasty was higher in Group B than in Group A but did not reach statistically significant difference (Fig. 1). There were no significant differences in adverse events or flexion degree between the groups (all *p* > 0.05) (Table 2).

## Discussion

This retrospective study compared two different injection treatments in obese patients with symptomatic, mild to moderate KOA. The patient groups were similar at baseline, with comparable KOA severity and PROM scores. Both groups reported reduced pain and improved function following injections; however, Group B regressed toward initial scores as follow-up time increased, whereas Group A remained less symptomatic than Group B and demonstrated better PROM scores at final follow-up compared to baseline. The most notable differences between the groups emerged at 6 months, 12 months, and final follow-up, when VAS and WOMAC scores were significantly lower in Group A (PRP) than in Group B (HA). Group B had significantly more arthroplasties than Group A (20 vs. 4), suggesting a higher rate of conservative treatment failure in the HA group. The Cox proportional hazards analysis showed only a non-significant trend toward a reduced risk of arthroplasty, and the Kaplan–Meier analysis did not demonstrate a statistically significant difference. Group A also demonstrated better knee extension ROM than Group B at 6 months and thereafter. The slightly improved knee extension may be attributable to reduced inflammatory activity and joint effusion, as limitations in full extension are generally more noticeable than limitations in full flexion in the presence of joint effusion. Although the observed difference was minimal, it reached statistical significance. However, its clinical relevance remains uncertain and can only be speculated upon. Overall, the results favor PRP injections over HA injections in terms of symptom relief and functional improvement. Group A underwent fewer arthroplasties than Group B; however, the Kaplan–Meier analysis did not demonstrate a statistically significant difference, showing only a non-significant trend toward a reduced risk of arthroplasty.

There was no significant difference in adverse events between the groups. The adverse events observed consisted of mild pain or swelling after injection, which are typical following intra-articular injections. Reported adverse event symptoms lasted for few days at maximum and vanished without issue. No signs of infection or other complications were detected. According to electronic medical records the patients with assumed adverse events did not have further contact with healthcare regarding the adverse events. The use of electronic medical records may result in underreporting of minor adverse events, as patients may disregard mild symptoms and not seek medical attention. Therefore, the incidence of adverse events may be underestimated. Nevertheless, such events are likely to be of limited clinical significance. Criteria to consider arthroplasty were KL grade 3 or 4 KOA, continuous symptoms for at least 6 months and failed extensive non-operative treatment. Patients who received PRP or HA injections typically had already failed basic conservative treatment of pain medication and PT and thus were sent for evaluation to the department of orthopedics for their KOA. No strict limit for WOMAC or VAS for pain was issued or if such limit was, then it is unknown to us due to the retrospective nature of this study.

A recent meta-analysis of double-blinded randomized controlled trials (RCTs) comparing PRP and HA in symptomatic KL 1–3 KOA showed that PRP is superior to HA in WOMAC scores, with a significant difference in minimal clinically important difference values, supporting the results of this study [13]. Compared to placebo (saline solution), PRP demonstrated modest efficacy for up to 6 months in another meta-analysis of RCTs, further supporting the result that PRP may be useful in treating the obese subgroup of KOA patients [14]. There appears to be a dose-dependent improvement in PROMs for patients treated with PRP injections, as demonstrated by a recent study comparing saline to single and triple doses of PRP administered at 1-month intervals; the study showed progressively better pain relief with three doses compared to saline or fewer PRP injections [15]. This study setting mirrors the literature in both optimal dosing and treatment timing with respect to KL stage of KOA. Additionally, there appears to be no statistically significant difference in growth factor composition in PRP between patients with higher versus lower BMI, suggesting that PRP efficacy is likely similar regardless of BMI [16]. The clinical implications of these results are significant, as they provide evidence-based guidance for treatment selection in obese KOA patients who face increased surgical risks and generally poorer outcomes following arthroplasty. PRP injection therapy may serve as a valuable therapeutic option for selected obese patients who do not respond adequately to conventional conservative treatments, offering a bridge to delay arthroplasty until optimal weight loss or other risk modifications can be achieved.

The strengths of this study include comprehensive demographic data collection, generally comparable patient groups at baseline (KOA severity and PROMs), a long follow-up period, and a patient population representative of typical real-world KOA patients with significant obesity. The use of commercially available PRP kits and hyaluronic acid products ensures that the study is readily reproducible. PRP dosing of three injections is likely better than two injections, as studies suggest that fewer injections result in poorer outcomes compared with at least three injections [17]. Recent studies suggest that leukocyte rich PRP and leukocyte poor PRP yield similar results in the treatment of KL 1–3 KOA with series of three injections, with no significant differences between the products, so the leukocyte rich PRP in this study was adequate in terms of leukocytes [18, 19].

This study has several limitations that warrant consideration. First, the retrospective design introduces inherent selection bias and precludes definitive causal inferences. The absence of randomization and blinding of patients and clinicians may have introduced bias in treatment allocation and outcome assessment. Further, treatment allocation bias, i.e. which patient receives which treatment, may affect the results. Patients were given a choice of both treatments, and they could freely pick either one. Treatments routines of single injection versus three injections may carry enhanced placebo effect favoring PRP, but this is unavoidable as it cannot be circumvented even in blinded study setting without compromising with either the quality of PRP or the quality of HA, thus skewing the overall potential of the treatment. Second, the sample size was modest (*n* = 99), resulting in a post hoc statistical power of only 47.5% for the primary outcome measure, which increases the risk of type II error. The modest sample size and limited number of events imposed a limitation for the statistical analyses, which is why conventional group comparisons were utilized instead of more complex longitudinal models such as mixed-effects or repeated-measures analyses. This of course increases risk of type I error from multiple comparisons. However, as this is retrospective study it was impossible to have larger population for analysis as the search date ranges to acquire the study population were predetermined to include all the patients from the start of introducing PRP treatments until chosen time point. This included all the patients who had intra-articular PRP injections and had available data regarding follow-up. Further studies should include even larger populations to address this. Third, significant differences in mean age (7.2 years), smoking status and follow-up duration (4.8 months) between groups represent potential confounding variables that may have influenced outcomes in terms of regaining range of motion and perhaps the risk of arthroplasty. Smoking may lower the patients’ activity levels and certainly increase the risks for arthroplasty, therefore reducing the overall problems that KOA is causing the patients making them less likely to search for arthroplasty and in turn surgeons to less likely operate these patients due to higher risks for surgery and as patients ‘manage’ with their KOA. Younger population may regain ROM better than older population and younger population may not be offered arthroplasty as an option as readily as for older patients. To address the time-dependent outcomes, the Kaplan–Meier survival analysis and Cox proportional hazards modelling were included to reduce the risk for confusion. Nevertheless, the mean follow-up difference may influence the risk of arthroplasty. Fourth, the study population lacked morbidly obese patients (BMI ≥ 35 or ≥ 40), limiting generalizability to this highest-risk subgroup who would potentially benefit most from effective conservative interventions. Different levels of obesity may influence the treatment response and this is an important area for future research. Fifth, the retrospective data collection precluded comprehensive characterization of the PRP preparation, including precise platelet-to-white blood cell ratios, growth factor levels and cytokine profiles, which limits reproducibility and comparison with other PRP formulations. The PRP was prepared using a commercially available kit that has been standardized to produce PRP with previously described composition and characteristics. Sixth, the heterogeneity of KOA severity (KL 1 to 3) was chosen to represent KOA that is generally still treatable without arthroplasty, but it carries the limitation that milder KL 1 patients may regress back to being symptomatic regardless of the treatment, while patients with KL 3 edging to progress to KL 4 KOA may receive little to no benefits, thus polarizing the PROM scores. Both groups had similar mean KL (PRP 2.35 ± 0.6 vs. HA 2.21 ± 0.6, *p* = 0.299) and the baseline radiographic severity was comparable between groups, suggesting that differences in outcomes were not driven by systematic differences in KOA stage. Dropping out the mildest KL 1 KOA patients would not represent the real-life spectrum of conservatively treatable KOA patients, and as previously stated PRP is most suitable for mild-to-moderate KOA (KL 1 to 3). Furthermore, additional subgroup analyses were not suitable according to individual KL grades as they would be underpowered and potentially unreliable. Seventh, we identified five patients from the source data that had KOA KL grade IV rather than grade III. This imposes a limitation by increasing the heterogeneity and possibly affecting arthroplasty outcomes. Eighth, the WOMAC subscores were not obtainable for every patient, therefore, only the total scores were included in the final analysis. This unfortunately prevents further subscale analyses for WOMAC. Ninth, the study lacked information regarding previous treatments that patients may or may not have received, including prior knee surgery, weight loss interventions, and previous injection therapies. Potential fluctuations in patients’ BMI during the study period were also unknown. However, as the study period preceded the widespread use of modern weight loss medications, substantial changes in BMI during follow-up are unlikely. Finally, the absence of cost-effectiveness analysis prevents evaluation of the economic implications of PRP versus HA therapy in this population. PRP treatment may increase healthcare burden through more contacts due to higher number of injections than HA, but the nature of the contacts itself is not demanding or burdensome. The total costs of three versus one injection are not possible to evaluate in this study setting as there was no data available to do so. Patients in both groups did not experience any additional costs depending on the treatment modality as the treatments were given in publicly funded healthcare.

Despite these limitations, this study demonstrates statistically significant differences between treatment groups with comparable baseline characteristics and sufficient follow-up duration to capture both short-term and long-term outcomes. However, the clinical relevance of these differences remains uncertain, as the minimal clinically important difference was not established. The results align with existing literature supporting the superiority of PRP over HA [5–7, 13] and contribute novel evidence regarding PRP’s potential to delay arthroplasty in obese patients [8, 9]. Nevertheless, given the methodological constraints, these findings should be interpreted with appropriate caution. Large-scale, prospective, randomized controlled trials with longer follow-up periods, standardized PRP protocols, inclusion of morbidly obese subgroups, and comprehensive cost-effectiveness analyses are warranted to confirm the sustained clinical and economic benefits of PRP therapy and to fully establish its role in the treatment algorithm for obese KOA patients. While PRP and HA have been extensively studied as potential treatments for KOA, subgroup analyses have been lacking, and this study addresses that knowledge gap. Obese patients appear to benefit more from intra-articular PRP injections than HA injections and demonstrate a lower risk of arthroplasty. This is a clinically significant finding, as it provides guidance for clinicians making treatment decisions for obese KOA patients. These findings should be interpreted as applying only to patients with a BMI between 30 and 35, as the study lacked a sufficient number of patients with a BMI ≥ 35 or ≥ 40 to allow meaningful subgroup analyses and to support similar conclusions in patients with morbid obesity. As this knowledge gap narrows, future studies should include other patient subgroups, such as morbidly obese patients, and should incorporate cost–benefit analyses for different treatments as well as comparative evaluations of treatment modalities in postponing arthroplasty.

## Conclusion

This retrospective study suggests that intra-articular PRP injections may have superior outcomes compared to HA injections in obese patients with BMI ranging from 30 to 35, with mild to moderate KOA. Patients treated with PRP experienced greater pain relief, improved functional outcomes, enhanced knee extension ROM, and a lower rate of progression to knee arthroplasty than those treated with HA. Although the study was underpowered, these findings suggest that PRP may be a more effective conservative treatment option for this high-risk patient population, potentially delaying the need for surgical intervention and providing sustained symptom relief over time. Similar or prospective study with larger population is warranted to confirm or negate the results.

## Data Availability

Data available on request due to restrictions (legal reason). Upon written request due to institutional policy.

## Abbreviations

BMI: Body mass index
ET: Exercise therapy
HA: Hyaluronic acid
KL: Kellgren-Lawrence
KOA: Knee osteoarthritis
PROM: Patient-reported outcome measure
PRP: Platelet-rich plasma
PT: Physical therapy
RCT: Randomized controlled trial
ROM: Range of motion
VAS: Visual analogue scale
WOMAC: Western Ontario and McMaster Universities Osteoarthritis Index

## Declarations

### Declaration of AI and AI-assisted technologies in the writing process

The author(s) confirm that no AI or AI-assisted technologies were used in the preparation of this manuscript. All content was generated, reviewed, and edited solely by the authors, who take full responsibility for the accuracy and integrity of the work.
